# Molecular Dynamics Simulation Study on the Occurrence of Shale Oil in Hybrid Nanopores

**DOI:** 10.3390/molecules29020312

**Published:** 2024-01-08

**Authors:** Yujia Fang, Zhongxiao Li, Erlong Yang, Mingyu Sha, Shuling Song

**Affiliations:** 1 Key Laboratory of Enhanced Oil & Gas Recovery, Ministry of Education, Northeast Petroleum University, Daqing 163318, China; lzx1417568834@163.com; 2 No. 8 Operation Area of No. 1 Oil Production Plant, Daqing Oilfield Co., Ltd., Daqing 163255, China; 13104598536@163.com; 3 San Ji Branch of Sinopec Oilfield Equipment Corporation, Wuhan 430040, China; 15776554410@163.com

**Keywords:** shale oil, occurrence state, illite, kerogen, nanometer pore

## Abstract

The molecular dynamics simulation was used to simulate the influence of the composite wall stacking effect on shale oil occurrence. The kerogen-illite heterogeneous wall pore model was established to study the effects of temperature, pore size, and wall component ratio on the adsorption ratio and diffusion capacity of shale oil. The calculation results show that the fluid density distribution in the hybrid nanopore is not uniform. When the pore size increases, the proportion of the first adsorption layer to the total adsorption amount decreases rapidly, and the phenomenon of the “solid-like layer” of shale oil in small pores is more obvious. In addition, increases in temperature have little effect on the density peak of the first adsorption layer. With increases in organic matter content in the shale pore model, the diffusion coefficient of fluid decreases gradually, along with adsorption capacity. The influence of the irregular arrangement of kerogen molecules on the adsorption of shale oil is greater than the influence of surface roughness caused by illite on the adsorption.

## 1. Introduction

Shale oil refers to liquid hydrocarbons in source rock shale. It is rich in reserves, widely distributed, and is a hot field of unconventional resource exploration after shale gas [1,2]. Shale oil contains a variety of hydrocarbon components. Shale reservoirs are characterized by high organic matter content and complex mineral types. The complex interactions such as adsorption and desorption between hydrocarbon components and mineral pore walls have a significant impact on the phase state of reservoir fluid [3]. Fluid exists in various forms in shale, and shale oil mainly exists in the form of adsorption or free-state. Under the current technical conditions, the movable reserves of shale oil are only 3~7%, and the free shale oil is the most recoverable part. Theoretically, the free shale oil content is the maximum available amount of shale oil, and it usually occurs in inorganic pores and fractures.

The occurrence form, occurrence state, and the difference of occurrence amount in different states of oil and gas have a very important influence on the dynamic process of oil and gas migration and the accumulation and the distribution of oil, gas, and water, which in turn affects the resource amount and mobility evaluation of the oil and gas exploration and development process. Understanding the characteristics of fluids such as oil in nanopores is conducive to the development and utilization of unconventional reservoirs, but micro–nano scale research is very difficult [4]. Some researchers have studied the properties of methane adsorption on different rocks through adsorption/desorption experiments, but few experiments have studied the properties of shale oil in nanopores, especially the effects of different rocks on shale oil adsorption.

Through the experimental study of organic matter, clay minerals, feldspar, quartz, calcite, dolomite, and other major organic/inorganic minerals, it is shown that the oil adsorption capacity is organic matter > clay minerals > felsic minerals (feldspar > quartz) > carbonate minerals (dolomite > calcite) [5]. Therefore, a large amount of adsorbed (including dissolved) shale oil mainly occurs in organic matter, and whether the adsorbed shale oil can be effectively exploited and developed directly affects the mobility, exploitation potential, and ultimate recovery of shale oil. Clay minerals account for a large proportion of the composition of shale, and clay minerals will have an important impact on the storage and migration of shale reservoirs. Wang et al. used molecular dynamics simulation to study the adsorption behavior of oil in carbonaceous crevices. The results show that the fluid density is unevenly distributed throughout the pore and oscillation is weakened from the pore surface to the central plane, which indicates that there are obvious adsorption layers and bulk fluids. They also studied the effects of pore size, temperature, pressure, and oil composition on the volume and density of the adsorption layer. It was found that liquid hydrocarbons always have multiple adsorption layers. The number of adsorption layers depends largely on the size of the slit and the composition of the oil. The adsorption tendency of heavy hydrocarbons is more obvious [6]. Lu et al. measured the adsorption capacity of mudstone shale samples and pure illite and concluded that although TOC is the primary influencing factor of shale adsorption capacity, the adsorption capacity of illite cannot be ignored, especially in shale reservoirs with less TOC [7]. Yang et al. used molecular dynamics to simulate the adsorption behavior of multi-component shale oil kerogen slits. The adsorbed asphaltene layer reduces the slit width and prevents the light component from being adsorbed on the kerogen slit due to the energy barrier formed by the heavy component. It is found that with the increase in temperature, the distribution of hydrocarbons is more uniform [8].

Molecular dynamics simulation results show that oil will adsorb to form a “solid-like layer” at the wall of nanopores, and the oil adsorption capacity of organic matter is much higher than that of clay minerals. A nuclear magnetic resonance test can be used to characterize pore size characteristics and the evolution of shale oil in different lithofacies. Studies have shown that the lower limits of pore size of adsorbed oil, free oil, and movable oil are 5 nm and 30~50 nm, respectively [9,10]. The fluid–solid interaction has a great relationship with the occurrence state of shale oil. The stronger the reservoir lipophilicity, the greater the adhesion of oil on its surface. The greater the adsorption thickness is, the smaller the proportion of free fluid is, and the smaller the available amount is.

The occurrence state of fluid in shale composite nanopores during production is the key to improving shale oil recovery [11]. In addition, pore types composed of inorganic minerals and organic matter of organic-rich shale are complex, and the pore scales are different. The types and sizes of interfacial forces at the nanoscale are also different. Based on shale oil reservoir characteristics, mobility, and production development status, the most effective way to improve shale oil recovery is to improve the reservoir’s physical properties, increase porosity and permeability, improve mobility, so that the occurrence and dissolved crude oil can flow, and reduce the viscosity of high-viscosity crude oil. In order to increase the production of shale oil, illite is one of the main components of many shale reservoirs [12]. Understanding the adsorption and desorption behavior of shale oil in illite is crucial for shale oil development. The desorption behavior of shale oil in illite nano-slits and its microscopic desorption mechanism are still unclear. Clay minerals account for a large proportion of the composition of shale, and clay minerals will have an important impact on the storage and migration of shale reservoirs. The pore volume and specific surface area of shale increase with the increase of illite content, and the presence of illite enhances pore surface roughness and pore heterogeneity. The adsorption of fluid molecules in the computational model is divided into two types, one is generated by the interaction between the pore surface and the fluid molecules, and the other is generated by the interaction between the fluid molecules, also known as the stacking effect [13]. It has been confirmed that when the pore size reaches or approaches the micro–nanometer level, the strong interaction between the pore wall and the fluid will affect the phase characteristics of the fluid, and the adsorption of fluid on the pore wall will affect the pore radius [14]. The different physical properties of inorganic and organic matter in shale bring different pore wall–fluid interactions, so their distribution cannot be ignored [15].

At present, the research on shale oil adsorption is mainly on the single-pore wall component, but in addition to organic matter, the actual reservoir also contains clay minerals and other components. In order to further approach real reservoir conditions, this study considers both organic matter and illite clay mineral, and mixing potential is used to distribute the force field. Through the study of the occurrence mechanism, the proportion of different occurrence states (adsorption and dissociation) can be inferred, which provides a theoretical basis for the mutual transformation conditions and quantitative characterization methods.

## 2. Models and Methods

### 2.1. Fluid Model under Composite Pores

The shale matrix is composed of inorganic substances and organic matter. The organic matter is mainly kerogen [16]. Kerogen is a sedimentary organic matter that generates oil and natural gas. It is an insoluble macromolecule dispersed in sedimentary rocks. Inorganic matter consists of minerals such as quartz, dolomite, calcite, and illite, of which illite is the major component. In previous studies, graphene was often used to characterize organic matter. Graphene molecules only contain carbon atoms and has an uncharged and very smooth surface, which leads to the excessive adsorption of shale oil and gas on the graphene wall. At the same time, the flow rate of shale oil in the pores of graphene is too high, which is quite different from the actual situation. Compared with graphene, the surface of the kerogen nanopores constructed in this paper is charged, containing five elements of carbon, hydrogen, oxygen, nitrogen, and sulfur, and the pore surface has obvious roughness, which is closer to the actual situation. This section combines the two components and uses kerogen and illite as the pore wall to construct a composite pore model to study the occurrence state of single-component shale oil in composite pores.

**Figure 1:**
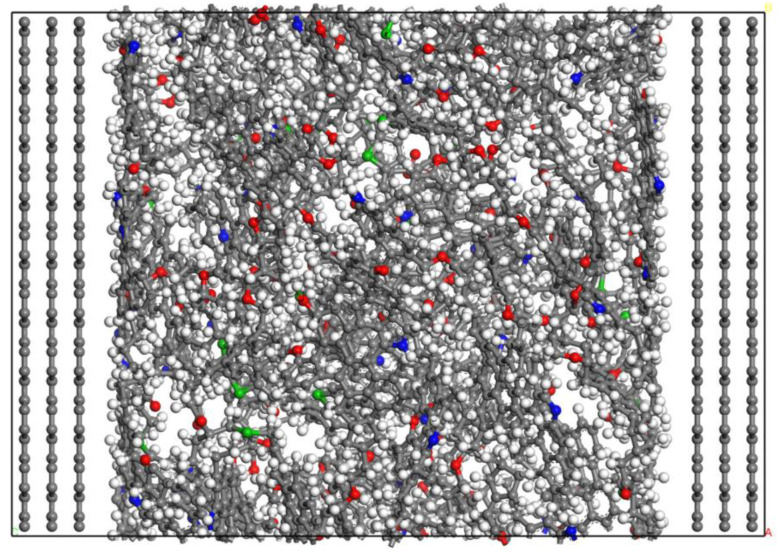
Kerogen aggregate model at t = 500 ps (Components color scheme: O, red; H, white; S, green; N, blue; C, grey).

**Figure 2:**
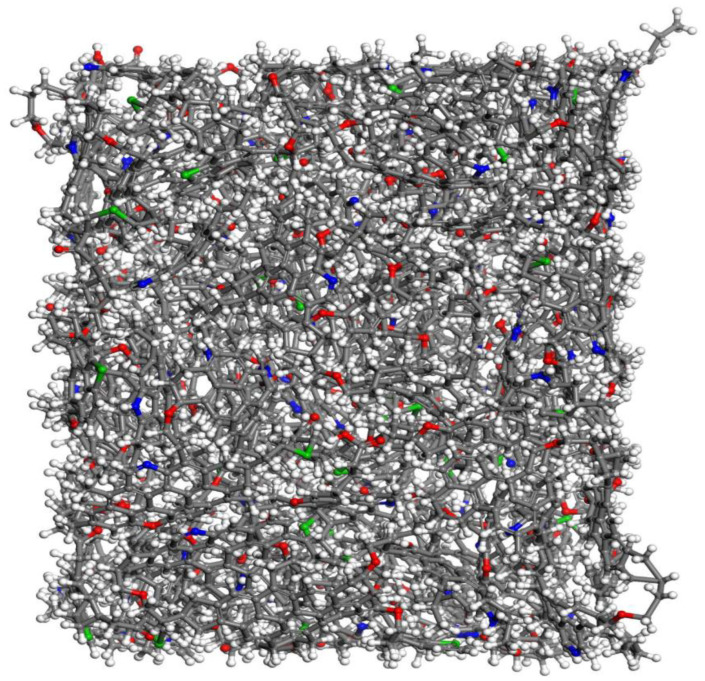
Compacted kerogen wall model (Components color scheme: O, red; H, white; S, green; N, blue; C, grey).

This section uses the kerogen structure designed by Ungerer et al. [17], which is based on the experimental results and the determination of element content and functional groups to obtain II-C kerogen, as it was oil-prone and corresponded to the kerogen in organic-rich shales. As it is enriched in organic shale [18], the characteristics of the organic matter may strongly differ depending upon the organic matter type and maturity [19]. The chemical formula of kerogen is C_242_H_219_O_13_N_5_S_2_ and it is not easy to combine it with the illite crystal structure because kerogen is a large aggregate. Therefore, kerogen should be compacted. Firstly, 19 II-C kerogen molecules were added into the pores composed of graphene sheets by Materials Studio 2020 software. After energy minimization treatment, the kerogen aggregates model was compacted by 500 ps relaxation treatment at 353 K and 30 MPa using an NPT ensemble. As the relaxation time increases, the kerogen aggregates are continuously compacted. However, due to the large kerogen molecules, the kerogen aggregates are not completely compacted, and there are still many pores, as shown in Figure 1. After that, the kerogen system was heated after 500 ps relaxation, and the whole system was simulated by an NPT ensemble at 773 K and atmospheric pressure. The simulation time was 2 ns so that the pores in the system were completely filled by sufficient thermal movement from the kerogen molecules. Then, the system was cooled and pressurized, and the whole system was simulated by an NPT ensemble for 2 ns under the temperature and pressure conditions of 353 K and 30 MPa. The final compacted kerogen is shown in Figure 2, and its size was 4.7 nm × 4.8 nm × 3.6 nm.

**Figure 3:**
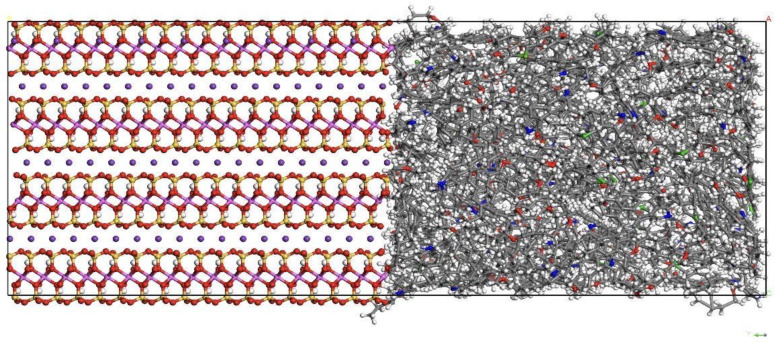
Composite pore wall model (Components color scheme: O, red; H, white; S, green; N, blue; Si, yellow; Al, pink; K, purple; C, grey).

In order to combine the kerogen wall model with the illite crystal structure, to ensure the same size as the adjacent surface, it is necessary to expand the illite crystal cell (9 × 6 × 4) to obtain a cell with a size of 4.7 nm × 4.5 nm × 3.6 nm and then combine it with the kerogen aggregates to obtain a pore wall composed of kerogen and illite. As shown in Figure 3, its size is 4.7 nm × 9.3 nm × 3.6 nm. The ratio of illite to kerogen is 1:1.

**Figure 4:**
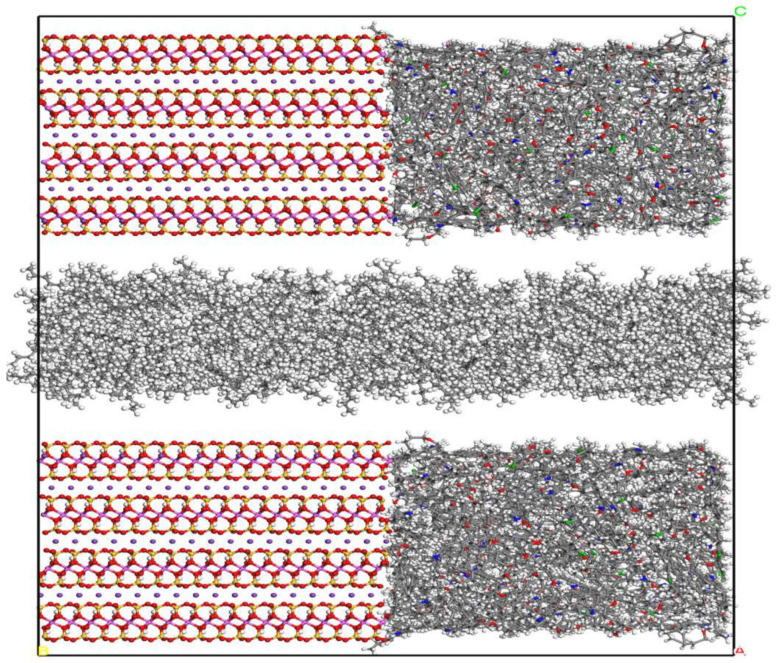
Fluid model under composite pores (Components color scheme: O, red; H, white; S, green; N, blue; Si, yellow; Al, pink; K, purple; C, grey).

In order to construct the fluid model, 402 n-octane molecules are added to the box with the same size as the wall in the XY direction to obtain a fluid box with an initial pore size of 2 nm. Combined with the composite pore wall, the fluid model of single-component shale oil under composite pores is obtained, as shown in Figure 4, and its size is 4.7 nm × 9.3 nm × 11.92 nm.

### 2.2. Simulation Method

The OPLS-AA (Optimized Potentials for Liquid Simulation) all-atom force field is used to describe the n-octane molecules (C_8_H_18_) in the pores. The potential energy parameters between different atoms in this force field are obtained by fitting the indoor experimental results, so it has high reliability. This force field is widely used in alkanes, polymers, biological macromolecules, etc. [20,21]. The potential energy parameters of C_8_H_18_ are shown in Table 1, Table 2, Table 3 and Table 4. C_3_, C_2_, and H are the carbon atoms and hydrogen atoms in alkanes, respectively.

Because the kerogen-illite wall is used, the mixing potential is used to distribute the force field. The potential energy parameters of atoms in kerogen are derived from the CVFF force field [22,23]. The potential energy parameters of atoms in illite are derived from the CLAYFF force field, and the Lorentz-Berthelot mixing rule is applied to the interaction between atoms of different molecules. The interaction between the T:O:T clay structure and cations is described by the CLAFF force field, which is widely used in clay interface simulation [24,25]. The CLAFF force field only considers the bond stretching and bond angle bending terms between water molecules, hydroxyl groups, soluble polyatomic molecules, and ions, and all other interaction forces are described by the sum of the non-bond interaction potential, namely the Lennard-Jones potential and the Coulomb force.

This simulation uses the large-scale parallel atomic operation simulator LAMMPS software (LAMMPS 3 Mar 2020) to simulate [26]. The temperature of the simulation system is controlled by the Nose–Hoover thermostat and set to 353 K. The periodic boundary is used in all three directions. The van der Waals interaction uses the Lennard-Jones potential, and the cutoff radius is 1.2 nm. The electrostatic interaction uses the PPPM algorithm with an accuracy of 10^−6^ [27]. The Lorentz-Berthelot mixing rule is used for the interaction between different molecules. The time step of the simulation is 1 fs, and 4 ns is simulated under the NVT ensemble to make the system reach equilibrium. If the statistical mean values of energy, temperature, and pressure of the system did not change with time, we considered that the system had reached equilibrium. The last 1 ns of time was taken and the data were collected at a 1 ps time interval for statistical analysis.

## 3. Occurrence Law of Shale Oil in Composite Pores

### 3.1. Occurrence Law of Shale Oil in Composite Pores

**Figure 5:**
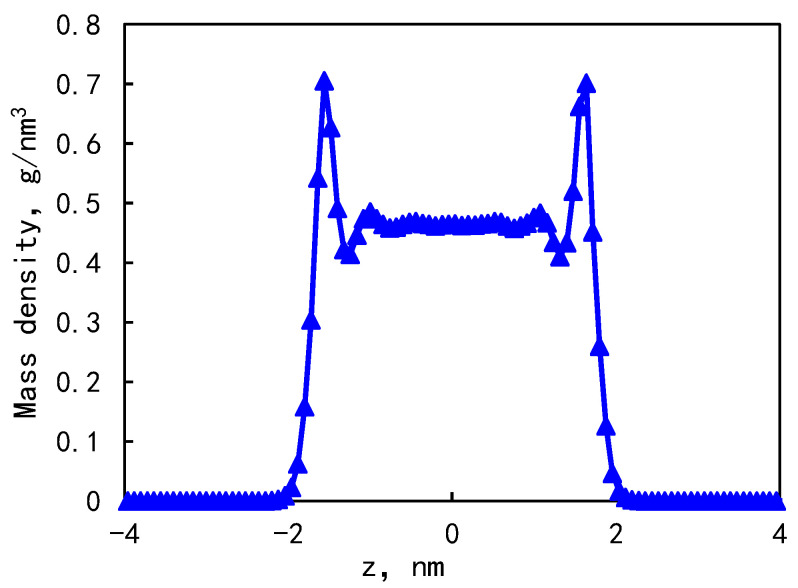
Distribution of fluid mass density in composite pores.

At a pressure of 30 MPa and a temperature of 353 K, the mass density distribution in the 5.36 nm composite pore is shown in Figure 5. The fluid mass density distribution combines the density distribution characteristics of kerogen and illite pores. The shale oil density distribution in the nanopores is uneven and symmetrically distributed along the center of the pores. There are two adsorption peaks on both sides of the wall, and the mass density is 0.71 g/cm^3^. The average density of the fluid in the bulk phase is 0.46 g/cm^3^. The density peak of the fluid in the first adsorption layer is 1.54 times that of the fluid in the bulk phase. Therefore, it can be considered that the adsorption layer exists in a solid or ‘solid-like’ form [28], as this phenomenon has been found in shale gas reservoirs [20]. After statistical calculation, the proportion of the adsorption phase is 31.25%. The thickness of the two adsorption layers is 0.5–0.6 nm.

The greater the interaction force between the n-octane molecule and the wall at the adsorption layer, the greater the density peak is, and the strong liquid-solid interaction near the wall leads to a large fluctuation amplitude of density. The closer to the center of the pore, the more stable the fluctuation amplitude is. This is because the peak size is related to the molecular arrangement law. The uneven distribution of atoms in the kerogen matrix leads to different interaction forces between wall atoms and n-octane at different positions, thus affecting the distribution of n-octane molecules on the pore surface. There are also such problems in the actual kerogen organic matter, so such a structure can better reflect the actual situation.

**Figure 6:**
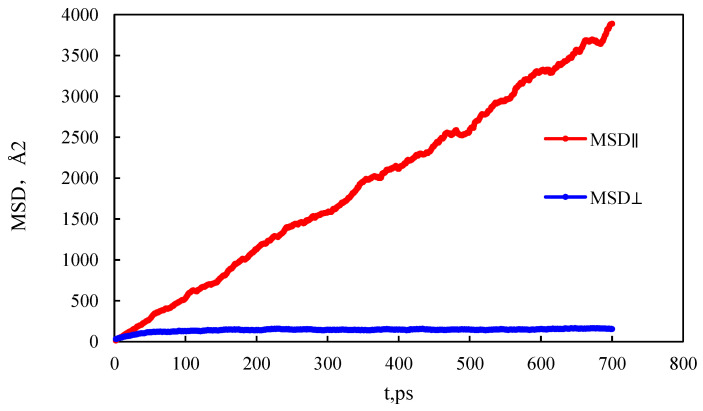
Mean square displacement curve.

During the simulation process, shale oil molecules will move irregularly, and the diffusion ability of shale oil molecules is related to solid–liquid interactions and interactions between shale oil molecules. The physical meaning of mean square displacement (MSD) is to count the average motion state of atoms in a certain period of time. According to the definition of the function expression, as shown in Formula (1), the calculation of mean square displacement can not only produce the statistical average from the number of atoms but also the sample average in a period of time, which makes the calculation of simulation results more stable and more accurate. As shown in Figure 6, the mean square displacement of shale oil molecules parallel to the wall is significantly higher than that perpendicular to the wall.

(1) $$MSD(t)=\langle {\left(x-x_{0}\right)}^{2}\rangle =\frac{1}{N}\sum_{n=1}^{N}{\left(x_{n}\left(t\right)-x_{n}\left(0\right)\right)}^{2}$$

where *N* is the average number of particles, $x_{n}(t)=x_{0}$ is the reference position of each particle, and $x_{n}\left(t\right)$ is the position of each particle at time *t*.

The diffusion coefficient can characterize the diffusion ability of the molecule. Combined with Formula (2), it can be calculated that the diffusion coefficient of shale oil molecules in the parallel wall direction is 0.90 × 10^−8^ m^2^/s, which is stronger than the diffusion coefficient of shale oil molecules in the vertical wall direction, 1.13 × 10^−16^ m^2^/s. This shows that shale oil molecules diffuse mainly in a way that is parallel to the wall.

(2) $$D=\frac{1}{2d}\underset{t\to \infty }{\lim}\frac{\langle {\left(x-x_{0}\right)}^{2}\rangle }{t}$$

where *D* is diffusion coefficient; *d* is the dimension of the simulation system.

### 3.2. Effect of Pore Size on Occurrence Characteristics

**Figure 7:**
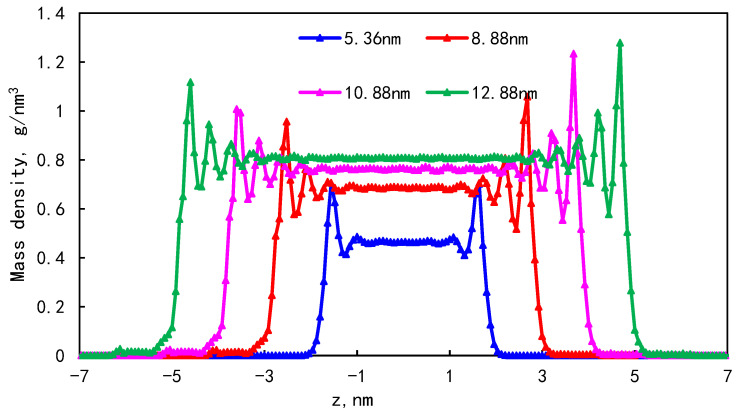
Density distribution in pore sizes of different kerogens.

In order to study the effect of pore size on the adsorption of single-component shale oil, models with pore sizes of 5.36 nm, 8.88 nm, 10.88 nm, and 12.88 nm were established under conditions of 353 K and 30 MPa. The mass density distribution of shale oil molecules under different pore sizes is shown in Figure 7. It can be seen that when the pore size is 5.36 nm, two adsorption layers are formed on both sides of the pore wall. In the pores with a pore size greater than 8.88 nm, four adsorption layers are formed on both sides of the wall. This is because when the pore size is small, the shale oil is greatly affected by the wall force and the solid–liquid force is strong. For larger pores, the interaction force between solid and liquid gradually weakens, and free fluid appears in the center of the pore. The more regular the molecular arrangement, the greater the peak value of adsorption number density. Because the kerogen wall is rough and porous, when shale oil molecules are arranged on the kerogen surface, there is a certain inclination angle with the XY plane with the fluctuation of the kerogen surface, which is the reason for the asymmetric distribution of the adsorption layer when adsorbed on the composite pore surface.

The proportion of the adsorption phase under different pore sizes is shown in Table 5. As the pore size increases, the bulk phase area increases and the proportion of the first adsorption layer to the total adsorption amount decreases rapidly, from 88.88% to 34.19%. In addition, the phenomenon of the “solid-like layer” of shale oil in small pores is more obvious. The larger the pore width, the larger the pore volume, but the adsorption capacity tends to remain unchanged and the proportion of adsorbed oil shows a downward trend. Small pores have a larger specific surface area, thus forming a larger adsorption wall. The storage capacity of small pores and large pores at a certain temperature reflects the proportion of free shale oil and the adsorbed state in pores with different pore sizes.

### 3.3. Effect of Temperature on Occurrence Characteristics

**Figure 8:**
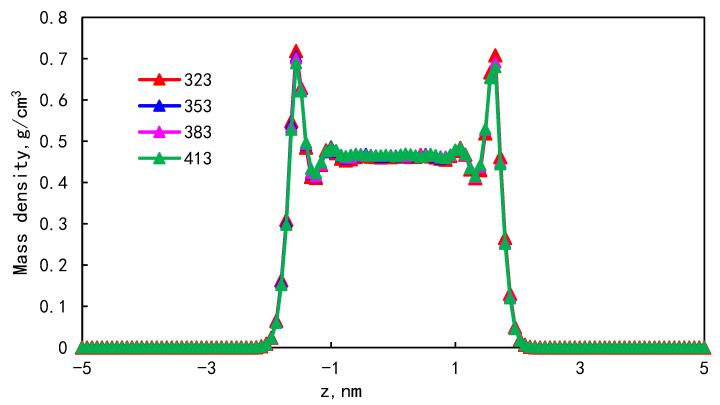
Density distribution at different temperatures.

In order to study the effect of temperature on the occurrence of single-component shale oil, the mass density distribution of shale oil molecules in shale composite nanopores at 323 K, 353 K, 383 K, and 413 K is shown in Figure 8. As the temperature increases, the density peak of the first adsorption layer decreases from 0.718 g/cm^3^ to 0.689 g/cm^3^, and the bulk density increases from 0.461 g/cm^3^ to 0.466 g/cm^3^. The results show that the increase in temperature has little effect on the density peak of the first adsorption layer.

The proportion of the adsorption phase at different temperatures is as shown in Table 6. During the temperature increase from 323 K to 413 K, the proportion of the adsorption phase decreases from 35.36 to 34.97%, and the proportion of the boundary layer adsorption phase decreases with the increase in temperature. The increase in temperature will reduce the interaction force between shale oil molecules and the wall surface, resulting in the desorption of molecules adsorbed on the wall surface. This shows that when the reservoir temperature increases, it is beneficial to the desorption of shale oil adsorbed on the pore wall, and the available shale oil in the bulk phase area increases, which has a positive effect on the exploitation of shale oil. In the process of shale oil development, increasing the reservoir temperature on the one hand can inhibit the adsorption of shale oil on the pore wall, on the other hand, due to the increase in temperature, the activity of fluid molecules is more active, which makes the fluid viscosity lower and easier to flow.

**Figure 9:**
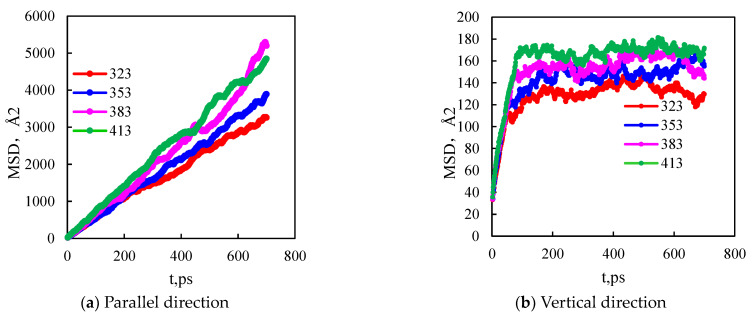
Mean square displacement at different temperatures.

The interaction energy of the liquid–solid system mainly includes van der Waals energy and electrostatic energy, in which the electrostatic energy is only related to the charge of the component atoms and is independent of temperature. The van der Waals energy of the system is calculated as a function of temperature. When the temperature of the system is 323 K, the van der Waals energy is −411.05 Kcal/mol. When the temperature rises to 383 K, the van der Waals energy is 555.68 Kcal/mol. As shown in Figure 9, the decrease of van der Waals energy in the temperature-rising system leads to the enhancement of the diffusion ability of alkane molecules in the direction perpendicular to the wall and a decrease in molecular adsorption. The implementation of thermal oil recovery technology in the field can effectively improve the recoverable reserves of shale and improve the final recovery rate.

### 3.4. The Effect of Wall Component Ratio on Occurrence

**Figure 10:**
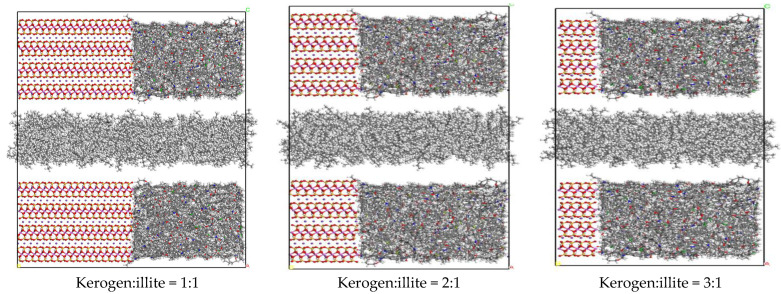
Fluid model with three different wall compositions (Components color scheme: O, red; H, white; S, green; N, blue; Si, yellow; Al, pink; K, purple; C, grey).

In order to compare the effects of different wall composition ratios on the occurrence of single component shale oil, three pore models with ratios of 1:1, 2:1, and 3:1 of kerogen:illite were established. The structural diagrams of three different wall compositions are shown in Figure 10.

**Figure 11:**
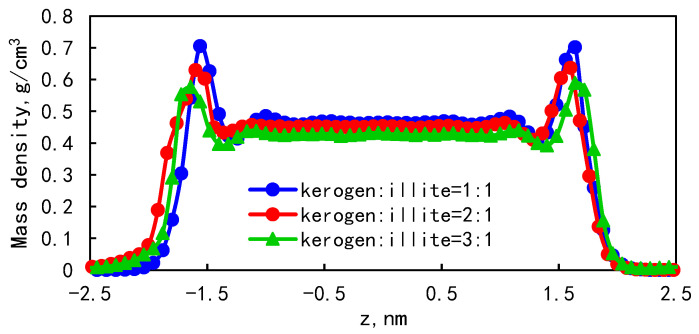
The mass density distribution of fluid under different wall composition ratios.

The mass density distribution of the fluid under different wall composition ratios is shown in Figure 11. When the ratio of kerogen to illite in the model wall is 1:1, the peak adsorption density is 0.71 g/cm^3^, and the adsorption phase ratio can reach 31.25%. When the ratio of kerogen to illite in the model wall is 3:1, the peak of adsorption density is 0.58 g/cm^3^, and the proportion of the adsorption phase is 41.71%. It is found that the higher the proportion of organic matter in the wall, the greater the adsorption capacity is. In the combined wall model, due to the difference in the binding energy between the wall and the fluid, the kerogen is more lipophilic, resulting in easier retention of shale oil and a larger adsorption ratio. It is interesting that the peak of adsorption density is positively correlated with the flatness of the wall. Therefore, the higher the kerogen content, the lower the flatness of the wall, and the smaller the density peak. The molecular arrangement of kerogen is irregular, and the specific surface area of the pore increases with the increase in illite content, which enhances pore surface roughness and pore heterogeneity. We also notice that the effect of relative kerogen molecular arrangement on shale oil adsorption is greater than that of surface roughness caused by illite.

**Figure 12:**
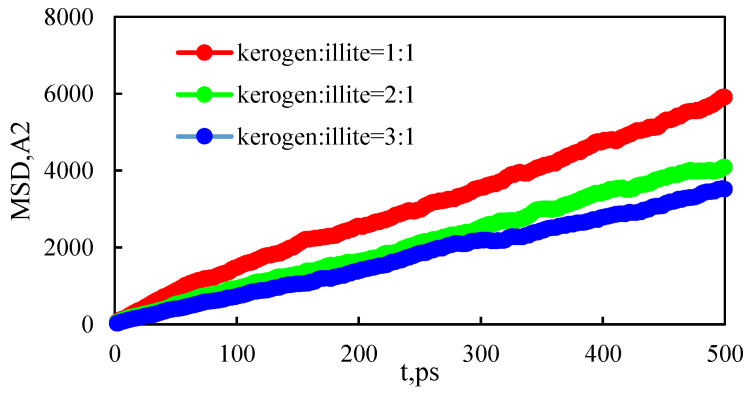
Mean square displacement of different wall component ratios.

In order to further characterize the effect of the wall component ratio on adsorption, the mean square displacement of different wall component ratios is shown in Figure 12. The mean square displacement of the model with kerogen:illite = 1:1 is higher than that of the model with kerogen:illite = 3:1. When the ratio of kerogen to illite in the model wall is 1:1, 2:1, and 3:1, the diffusion coefficients were 1.84 × 10^−10^ m^2^/s, 1.28 × 10^−10^ m^2^/s, and 1.03 × 10^−10^ m^2^/s, respectively. This demonstrates that the diffusion ability is enhanced with the increase in the proportion of organic matter in the wall. With the increase in organic matter content in shale pore model, the diffusion coefficient of fluid decreases gradually. In addition, it can be found that the content of organic matter in shale has a significant negative effect on the self-diffusion performance of shale oil in nanopores.

Since the diffusion capacity is related to the solid–liquid interaction and friction coefficient, the friction coefficient is related to the roughness of the wall. In this case, the combined action of the solid–liquid interaction force and friction force makes shale oil more likely to occur in a wall with a high proportion of organic matter.

## 4. Conclusions

(1) The mass density distribution of the composite wall was analyzed. The results show that the density distribution of octane is not uniform, showing multi-layer adsorption. The thickness of the two adsorption layers is 0.5–0.6 nm, which is the reason for the asymmetric distribution of the adsorption layer when adsorbed on the composite pore surface. The mean square displacement of shale oil molecules parallel to the wall is significantly higher than those perpendicular to the wall, showing that shale oil molecules diffuse mainly in a way that is parallel to the wall.
(2) As the pore size increases, the bulk phase area and pore volume increase but the adsorption capacity tends to remain unchanged, causing a downward trend in the proportion of adsorbed oil. In addition, the proportion of the first adsorption layer to the total adsorption amount decreases rapidly, from 88.88% to 34.19%, and the phenomenon of the “solid-like layer” of shale oil in small pores is more obvious.
(3) The higher the proportion of organic matter in the wall, the greater the adsorption capacity. Because kerogen is more lipophilic, shale oil is more easily retained and the adsorption capacity is larger. The peak of adsorption density is positively correlated with the flatness of the wall. When the ratio of kerogen to illite in the model wall is 1:1, 2:1, and 3:1, the diffusion coefficients were 1.84 × 10^−10^ m^2^/s, 1.28 × 10^−10^ m^2^/s, and 1.03 × 10^−10^ m^2^/s, respectively. The combination of solid–liquid interaction force and friction force makes shale oil more likely to occur in the wall with a high proportion of organic matter.

## Figures and Tables

**Table 1:** Non-bonded potential energy parameters of an n-octane molecule.

	Atomic Type	Molar Mass/ g/mol	σ /Å	ε /kcal/mol	Electric Charge /e
Non-bonded potential energy	C_3_, C_2_	12.0112	3.50	0.066	−0.18, –CH_3_ −0.12, –CH_2_
	H	1.0080	2.50	0.030	0.06

**Table 2:** Bond stretching energy parameters of an n-octane molecule.

	Bond Type	$K_{r}$, kcal/(mol·Å^2^)	$r_{0}$, Å
Bond stretching	C_2,3_–C_2,3_	268.00	1.529
	C_2,3_–H	340.00	1.090

**Table 3:** Angle bending energy parameters of an n-octane molecule.

	Key Angle Type	$K_{\theta }$, kcal/(mol·radian^2^)	$\theta_{0}$, Radian
Angle bending	C_2,3_–C_2,3_–H	37.50	1.932
	H–C_2,3_–H	33.00	1.881
	C_2,3_–C_2,3_–C_2,3_	58.35	1.967

**Table 4:** Dihedral angle energy parameters of an n-octane molecule.

	Dihedral Angle Type	$C_{1}$, kcal/mol	$C_{2}$, kcal/mol	$C_{3}$, kcal/mol	$C_{4}$, kcal/mol
Dihedral angle	H–C–C–C	0.0	0.0	0.366	0.0
	H–C–C–H	0.0	0.0	0.318	0.0
	C–C–C–C	1.740	0.159	0.279	0.0

**Table 5:** The proportion of the adsorbed phase under different pore sizes.

Pore size/nm	5.36	8.88	10.88	12.88
Proportion of adsorption phase/%	35.19	34.30	32.99	31.53
The proportion of the first adsorption layer/%	31.25	18.72	13.55	10.78
The ratio of the first adsorption layer to the total adsorption amount/%	88.80	54.58	41.07	34.19

**Table 6:** The proportion of the adsorption phase at different temperatures.

Pore size/nm	323	353	383	413
Proportion of adsorption phase/%	35.36	35.19	35.04	34.97
The proportion of the first adsorption layer/%	31.45	31.25	31.10	30.97
The ratio of the first adsorption layer to the total adsorption amount/%	88.92	88.79	88.75	88.56

## Data Availability

Data are contained within the article.
